# Effect of topiramate on interleukin 6 expression in the hippocampus of amygdala-kindled epileptic rats

**DOI:** 10.3892/etm.2013.1396

**Published:** 2013-11-08

**Authors:** FEI YE, XU-QIN CHEN, GUAN-SHUI BAO, YIN HUA, ZHE-DONG WANG, YI-CHUAN BAO

**Affiliations:** 1 Department of Neurology, No.3 People’s Hospital Affiliated to Shanghai Jiao Tong University School of Medicine, Shanghai 201900, P.R. China; 2 Department of Neurology, Affiliated Children’s Hospital of Soochow University, Suzhou, Jiangsu 215000, P.R. China; 3 Department of Child Neurology, Wuxi People’s Hospital, Wuxi, Jiangsu 214001, P.R. China; 4 Department of Neurology, Jinhua Municipal Central Hospital, Jinhua, Zhejiang 321000, P.R. China; 5 Xi’an Jiaotong-Liverpool University, Suzhou, Jiangsu 215123, P.R. China

**Keywords:** interleukin-6, epilepsy, kindling, rat, basolateral amygdale, topiramate

## Abstract

The objective of this study was to analyze the changes in expression and the possible functions of interleukin-6 (IL-6) in electrical kindling of the basolateral amygdala (BLA) in epileptic rats. Bipolar electrodes were implanted into the BLA of Sprague-Dawley rats, and the rats were then subjected to chronic electrical stimulation through the electrodes to induce kindling. The seizure characteristics and behavioral changes of the rats were observed, and electroencephalograms were recorded during and following kindling. The IL-6 mRNA expression in the hippocampi of the rats was analyzed using semi-quantitative reverse transcription-polymerase chain reaction, and control and topiramate (TPM)-treated groups were compared. The mean time-period required for kindling was 13.50±3.99 days, and the afterdischarge duration (ADD) measured between 21,450 and 119,720 msec. The expression of IL-6 mRNA was significantly upregulated in the kindled rats. TPM was able to depress the seizures and decrease the IL-6 level in the kindled rats. In conclusion, IL-6 mRNA was upregulated in the hippocampi of epileptic rats, and IL-6 may have participated in the process of kindling.

## Introduction

A number of studies have suggested that immune factors are involved in the pathogenesis of epilepsy (1–3). During seizures, the cellular immune function becomes abnormal, which leads to changes in the intracorporeal cytokine levels. Among these cytokines, interleukin-6 (IL-6), in particular, has been implicated. IL-6 is generally synthesized by mononuclear phagocytes, vascular endothelial cells and fibroblasts. It is also produced by activated T cells. The main biological function of IL-6 is stimulating the maturation of activated B cells. In the nervous system, neurons and glial cells (including microglia and astrocytes) all secrete IL-6. The hippocampus, neocortex and cerebella exhibit high expression levels of IL-6 and its receptor. Similar to other cytokines, IL-6 binds to the α chain of the IL-6 receptor (IL-6R) on cell surfaces. Subsequently, the α chain and the receptor subunit gp130 (IL-6Rβ chain) form a high-affinity receptor that transmits a signal into cells through the gp130/Jak/STAT route to regulate gene transcription (4). In general, IL-6 has a neuroprotective effect on ischemic lesions, glutamate excitotoxicity and tumor necrosis factor (TNF)-mediated injuries (5–7). Furthermore, IL-6 has been shown to promote neuronal survival. In a study by Thier *et al*(8), it was revealed that the IL-6 secreted by sensory nerves and the IL-6 receptors jointly acted as neurotrophic factors for the maintenance of neuronal survival. In addition, IL-6 has shown the ability to induce PC12 cells to differentiate into nerve cells (9). IL-6 is able to protect striatal neurons against *N*-methyl-D-aspartate (NMDA) toxicity (8) and dopaminergic neurons against 1-methyl-4-phenylpyridinium (MPP) toxicity (10). Moreover, IL-6 is able to promote the regeneration of severed peripheral nerve cells (11). März *et al*(12) demonstrated that IL-6 was able to induce cultured typical stellate cells to transform into fibroblast-like cells, and to induce the expression of neurotrophic factors. Moreover, in different brain regions, IL-6 induced different neurotrophic factors, thus indicating that IL-6 was involved in multiple central nervous system (CNS) functions (11). It has been suggested that IL-6 directly participates in the excitation of nerve cells. Tancredi *et al*(13) demonstrated that IL-6 inhibited the post-tetanic potentiation (PTP) and the long-term potentiation (LTP) of nerve cells in the hippocampal CA1 region, as well as participating in the plasticity of the hippocampal synapse. Therefore, IL-6 is closely associated with neurological excitability. Qiu *et al*(14) showed that increased IL-6 levels were capable of notably changing the response to excitatory neurotransmitter during CNS neuronal development, and that IL-6 pretreatment was able to change the level of influx of extracellular calcium ions and the release of stored calcium, thus selectively enhancing the response of intracellular calcium ions to NMDA. In an experiment with mice, IL-6 was shown to be involved in the response to cerebral injury (15). In addition, a number of studies have demonstrated that IL-6 is able to influence the ethological manifestations of animals (16–18). IL-6 has been suggested to be associated with stress (19), as well as certain nervous and mental illnesses, such as infantile autism (20) and migraine (21). Furthermore, IL-6 is closely associated with neurological excitability; thus it has been proposed that IL-6 may be involved in epilepsy. In 1999, Jankowsky and Patterson (2) observed increased levels of oncostatin M (OSM), leukemia inhibitory factor (LIF), cardiotrophin-1 (CT-1), ciliary neurotrophic factor (cNTF) and other cytokines in the hippocampi of pilocarpine-induced epileptic rats, and proposed that cytokines were possibly intercellular informational molecules in the mechanism underlying the formation of epilepsy. D’Arcangle *et al*(22) later demonstrated that IL-6 was able to reduce synapsin I phosphorylation, and transgenic mice with the synapsin I deletion have been shown to be susceptible to epileptiform seizures during growth (23). Therefore, IL-6 may be involved in epileptic formation. Steffensen *et al*(24) observed that although IL-6 had a certain protective effect on nerve cell damage under general conditions, the continuously high expression of IL-6 in transgenic mice increased neuron deficiency, gliosis and hippocampal seizure discharge, inhibited the θ rhythm and participated in epileptogenesis. Results from a number of studies (25–27) have indicated that convulsions caused by viral infections may be associated with IL-6. In a previous study, IL-6 was shown to aggravate thermal shock in young mice (28). Moreover, in a study by Lehtimäki *et al*(29), northern blotting and *in situ* hybridization were used to assess the effects of kainic acid on the levels of cytokines and their corresponding receptors in the CNS of rats, and it was revealed that IL-6 and IL-6R expression levels had increased. IL-6 has also been suggested to be involved in the control of epilepsy, as well as neuronal damage and protection. Kalueff *et al*(30) revealed that IL-6 was capable of aggravating chemical-induced rat convulsions. Furthermore, in a study by Peltola *et al*(31), it was observed in the clinic that, following an epileptic attack, IL-6 levels in the cerebrospinal fluid of patients appeared to increase. It was therefore proposed that IL-1, IL-6 and TNF were regulators of colloid cells following CNS damage. In addition, Lehtimäki *et al* observed that a one-time epileptic attack was capable of increasing the IL-6 concentration in the blood of patients (32). A Japanese study showed that among pediatric patients with influenza and neurological complications, the IL-6 concentration in the blood of the convulsion group was higher than that of the delirium group and the control group, respectively (33). Therefore, the interrelation between IL-6 and epilepsy requires further study. As clinical research is limited in the actual condition of patients, it is only possible to detect the expression of IL-6 in cerebrospinal fluid or blood. Furthermore, the expression of IL-6 in living brain tissue is unclear. However, the majority of the previous animal experiments have used a chemical-induced epileptic rat model, with which it was difficult to avoid the impact of the chemicals themselves on the toxicity and stimulatory effect of IL-6. Therefore, the present study used the electrical kindling rat model of epilepsy to explore the possible roles of IL-6 and antiepileptic drugs in the incidence and treatment of epilepsy. By observing the changes in the IL-6 mRNA expression level in the hippocampi of electrically kindled epileptic rats, and the effect of an antiepileptic drug on the rats, this methodology was able to avoid the direct toxic action of chemical drugs and reduce the interference of the chemicals, thus making any obtained conclusions more reliable.

Topiramate (TPM) is a second-generation antiepileptic drug that affects a variety of cellular mechanisms (34), such as the inhibition of voltage-dependent sodium channels and the negative modulation of L-type calcium channels. TPM has been suggested to exhibit neuroprotective and antiepileptic effects (35), and was therefore used in this study.

## Materials and methods

### Animals and grouping

A total of 24 specific pathogen-free (SPF) male Sprague-Dawley (SD) rats, with body weights ranging from 180 to 280 g, were obtained from the Experimental Animal Center of Soochow University (License no. SYXK-Su-2002-0037; Suzhou, China). The rats were randomly divided into four groups: blank control, surgical control, epilepsy and TPM, with six rats in each group. The rats were bred in separate cages away from intense light, under a 24-h circadian cycle, and were allowed to freely eat and drink. This study was performed in strict accordance with the recommendations in the Guide for the Care and Use of Laboratory Animals of the National Institutes of Health (8th edition, 2011). The animal use protocol was reviewed and approved by the Institutional Animal Care and Use Committee (IACUC) of Soochow University.

### Preparation of electrically kindled animal models

All rats, with the exception of those in the blank control group, were anaesthetized with 4% (mass concentration) chloral hydrate and fixed onto the brain stereotaxic apparatus. The incisor was set to 2.4 mm below the ear rod. Based on the spectra, the left basolateral amygdaloid nucleus of the rats was determined (2.0 mm caudal to the bregma, 5.0 mm lateral to the midline and 8.0 mm ventral to the dura). A bipolar electrode with a diameter of 0.20 mm was inserted and fixed onto the surface of the skull. The rats were left to recuperate for 1 week following the surgery, and were then stimulated with 2-sec-long 50-Hz, 400-μA continuous single-phase square waves with a width of 1 msec, once daily. For the blank control and surgical control groups, no stimulation was applied. Following Racines’s grading (36), the seizure intensities were observed and recorded. If the rats presented a grade 5 reaction five consecutive times, the kindling was deemed successful and stimulation was immediately stopped.

### Synchronous recording of a video electroencephalogram

A Cadwell video electroencephalogram analysis system (Cadwell Laboratories, Inc., Kennewick, WA, USA) was used to synchronously record the background electroencephalogram of the rats in the free state and the afterdischarge duration (ADD) during electrical stimulation. Afterdischarge (AD) refers to a period of epileptiform activity observed with at least five confirmable high-amplitude, multiple-spiked waves that appear following electrical stimulation. ADD is the duration from the end of stimulation to the end of the AD.

### Drug experiment and specimen acquisition

Following successful kindling, 2 ml/day normal saline was intragastrically administered to the rats in the blank control, surgical control and epilepsy groups, whereas 85 mg/kg/day TPM was intragastrically administered to the rats in the TPM group. For all groups, the intragastric administration was conducted for eight days. In the epilepsy and TPM groups, electrical stimulation was conducted once prior to the intragastric administration, according to the parameters required for kindling. The characteristics of the seizures and the corresponding electroencephalograms were recorded simultaneously. At the end of the intragastric administration, the electrical stimulation was repeated, and the resulting electroencephalograms were recorded. Moreover, the characteristics of the seizure and the changes in the electroencephalograms between the epilepsy and TPM groups were compared. Twenty-four hours subsequent to the second electrical stimulation, the rats were anaesthetized and the thoracic cavity was opened. Following the puncturing of the aortic arch, the brain of each rat was extracted and washed with phosphate-buffered saline (PBS) and the right cerebral hippocampi were homogenized. The homogenates were stored at −70°C for future use.

### Reverse transcription-polymerase chain reaction (RT-PCR)

The homogenized hippocampal tissues were treated with TRIzol^®^ reagent (Invitrogen Life Technologies, Carlsbad, CA, USA) for cell lysis, and then the RNA was extracted with chloroform, precipitated with isopropyl alcohol, eluted with ethyl alcohol and dissolved in diethyl pyrocarbonate (DEPC) solution. Following this, an ultraviolet spectrophotometer (752 Ultraviolet Spectrophotometer Model; Shanghai Precision Scientific Instrument Corp., Shanghai, China) was used to measure the absorbance at 260 nm (A_260_), and the RNA concentration was calculated. Subsequently, reverse transcription was performed and the cDNA product was stored at 4°C. The obtained cDNA was amplified using PCR with the following primers: β-actin (expected product size of 265 bp) forward primer (P1), 5′-AGGCCGGCTTCGCGGGCGAC-3′, and reverse primer (P2), 5′-CTCGGGAGCCACACGCAGCTC-3′; and IL-6 (expected product size of 442 bp) forward primer (P3), 5′-GAGAAAAGAGTTGTGCAATGGC-3′, and reverse primer (P4), 5′-ACTAGGTTTGCCGAGTAGACC-3′. The PCR conditions were as follows: initial denaturation at 94°C for 5 min, followed by 30 cycles of denaturation at 94°C for 30 sec, annealing at 56°C for 30 sec and extension at 72°C for 1 min, followed by a final extension step at 72°C for 7 min. Subsequent to electrophoresis, the PCR amplification products were analyzed using a gel imaging analysis system. A BioCaptMW analysis system (BioCaptMW version 99.05 s system; Vilber Courmat, Marine-La-Vallee, France) was used to detect the gray value of each band, and β-actin was used as the internal reference to semiquantitatively determine the IL-6 mRNA expression levels.

### Statistical analysis

The SPSS statistical software package (SPSS, Inc., Chicago, IL, USA) was used for all statistical analysis. The experimental data are expressed as the mean ± standard deviation. The means of the measurement data were compared among the multiple groups using one-way analysis of variance (ANOVA), once the homogeneity of the variance had been confirmed. The Student-Newman-Keuls (SNK) method was used for comparisons between groups. A Fisher’s test was used for the count data. P<0.05 was considered to indicate a statistically significant difference.

## Results

### Kindling and electroencephalogram records

**Figure 1:**
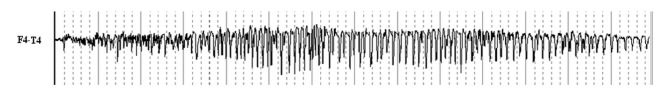
Discharge of epileptic seizures in the rat.

With regard to the rat kindling process, the shortest period required to achieve kindling was 7 days, while the longest period was 19 days. The mean number of days was 13.50±3.99 days. In the epilepsy group, all the rats presented with grade 5 seizures. Following TPM treatment (the TPM group), the intensity, amplitude and duration of the seizures were decreased, or the seizures of the rats were completely controlled. Among the TPM group, one rat exhibited grade 1 seizures, three rats exhibited grade 3 seizures and two rats exhibited grade 4 seizures. None of the rats exhibited grade 5 seizures in the TPM group. The two groups were significantly different with regard to seizure grade (P<0.05). At all recorded points, the ADD values of the electrically kindled rats were between 21,450 and 119,720 msec (Fig. 1). Moreover, the mean ADD values of the epilepsy and TPM groups were 78,205.67±32,567.93 and 23,880.83±20,184.50 msec, respectively. The ADD of the TPM group was significantly shorter than that of the epilepsy group (P<0.05).

### RT-PCR detection of IL-6

**Figure 2:**
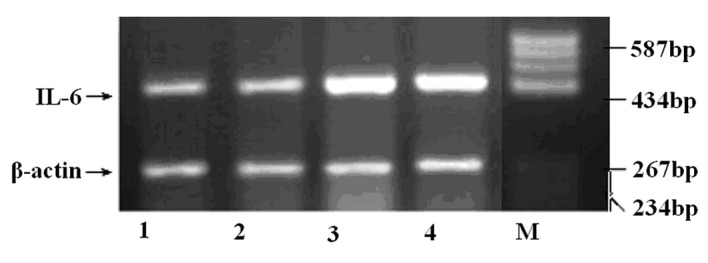
Electrophoresis of the reverse transcription polymerase chain reaction products of interleukin-6 (IL-6). Lane 1, blank control; lane 2, surgical control; lane 3, epilepsy; lane 4, topiramate (TPM); lane M: marker.

The variance analyses of the semiquantitative RT-PCR results for IL-6 mRNA in the hippocampi of the rats in the four treatment groups were significantly different (F=5.959, P<0.05). As shown in Fig. 2, compared with the levels in the two control groups (1.57±0.69 for the blank control group and 1.80±0.54 for the surgical control group), the IL-6 mRNA levels (mean 2.83±0.40) in the hippocampi of rats in the epilepsy group were significantly increased (P<0.05). In addition, the TPM group (2.18±0.56) had a different recovery rate; however, the difference in IL-6 expression was not significant between the TPM and epilepsy groups (P>0.05; Fig. 2).

## Discussion

As a model for temporal lobe epilepsy, the kindling model is widely used in studies of epilepsy, since the spontaneous limbic system epilepsy caused by electrical kindling is similar to temporal lobe epilepsy. In the CNS, a number of regions are able to act as electrical stimulation sites for kindling rats. Among these regions, the basolateral amygdaloid nucleus has the most extensive conduction bundle link, and may be the focus of the epileptiform manifestations in the limbic system (36). In the present study, a solid electrode was inserted into the rats, and electroencephalograms were recorded when the rats were in the free state. This surgical method was convenient, and the detection results and the actual values were reliable. Furthermore, compared with the chemical-induced epilepsy model, the surgical method avoided the interference of drug toxicity on the experimental data and improved the reliability of the results.

The mechanisms that regulate IL-6 expression in the hippocampus have yet to be elucidated. IL-1, interferon (IFN) and TNF, as well as certain neurotransmitters, are capable of promoting IL-6 secretion (37–39), and it was observed that following LTP induction with an embedded electrode, the expression levels of certain cytokines were notably increased. In particular, IL-6 levels were more significantly increased than those of the other cytokines (39). In a study on the changes in IL-6 expression following cerebral ischemia in rats, Ali *et al*(40) demonstrated that NMDA and ionomycin (a calcium ion carrier) upregulated IL-6 mRNA expression, which indicated that the NMDA receptor-mediated calcium ion influx induced the neurons to generate IL-6. Therefore, LTP and calcium ion influx induce neurons to generate IL-6. The present study demonstrated that the level of IL-6 expression in the hippocampi of electrically kindled rats was increased. One potential mechanism for this is that the electrical kindling increased the levels of excitatory neurotransmitters and decreased the levels of γ-aminobutyric acid (GABA) in the neurons, thereby increasing the influx of neuronal calcium ions, and, consequently, neuronal IL-6 expression. Furthermore, the LTP induced by electrical stimulation may also have been involved in promoting IL-6 secretion.

The involvement of IL-6 with nervous system diseases has been an area of increasing focus, and IL-6 is generally recognized to exhibit neuroprotective effects (41). However, compared with wild-type mice, the hippocampal seizure discharge rates of transgenic mice with continuously high IL-6 expression have been shown to be increased, whereas the θ rhythm of the transgenic mice was inhibited; this continuously high IL-6 expression has been indicated to be involved in seizure formation (24). In the present study, the IL-6 expression in the hippocampi of the electrically kindled rats was increased. The preliminary increase in IL-6 expression may have been a protective mechanism intended to preserve the neurons. However, the continuously high expression may have been involved in the kindling process by increasing gliosis and hippocampal seizure discharge, among other mechanisms (25). Antiepileptic drugs decrease IL-6 expression by varying degrees, and may therefore be useful for maintaining the appropriate IL-6 levels. TPM reduces the frequency and intensity of seizures in epileptic rats, in addition to reducing the cerebral injury caused by epilepsy or anoxia (42). In the present study, although TPM was able to induce the recovery of the IL-6 levels, the difference between the IL-6 levels in the TPM and epilepsy groups was not significant. This phenomenon may have been due to the relatively small sample size utilized, as well as insufficient control over the experimental conditions in the study. Thus, this result remains to be verified in future studies with larger sample sizes. Further studies are also required to investigate the exact mechanisms underlying the action of IL-6 in epilepsy.
